# Effect-Allele Frequencies at Cardiometabolic Risk Loci in an Uzbek Cohort from Tashkent: A Central Asian Reference and Priorities for Polygenic Score Validation

**DOI:** 10.3390/genes17091117

**Published:** 2026-09-15

**Authors:** Alisher A. Abdullaev, Darya V. Zakirova, Sergei A. Kosushkin, Fazliddin Z. Xonboev, Guzal J. Abdullaeva, Rano B. Alieva, Shahlo U. Turdikulova

**Affiliations:** 1 Center for Advanced Technologies, Talabalar Street, Tashkent 100174, Uzbekistan; daryazakirova89@gmail.com (D.V.Z.); toki.sk@gmail.com (S.A.K.); mr.khonboev@gmail.com (F.Z.X.); shahlo.ut@gmail.com (S.U.T.); 2 Republican Specialized Scientific and Practical Medical Center of Cardiology, Osiyo Street 4, Tashkent 100052, Uzbekistan; guzal-abdullaeva@bk.ru (G.J.A.); ranoalieva@mail.ru (R.B.A.)

**Keywords:** type 2 diabetes, allele frequency, Central Asia, Uzbekistan, Tashkent, population genetics, polygenic risk score, 1000 Genomes

## Abstract

Background/Objectives: Central Asian populations are almost entirely absent from genomic reference resources, yet Uzbekistan has one of the fastest rates of growth in age-standardised type 2 diabetes (T2D) incidence. Allele frequencies at cardiometabolic risk loci are unknown, limiting the interpretation of polygenic risk scores (PRS) derived elsewhere. We aimed to characterise effect-allele frequencies in an Uzbek cohort from Tashkent and quantify divergence from 1000 Genomes panels to guide future PRS evaluation. Methods: We genotyped 171 adults from Tashkent (131 with T2D, 40 without) on a custom Agilent SureSelect panel of cardiometabolic candidate SNPs. After quality control, 1939 variants were analysed. Effect-allele frequencies were compared with 1000 Genomes Phase 3 superpopulations (EUR, SAS, EAS, AFR) using Hudson FST and Nei’s genetic distance with bootstrap 95% CIs (1000 replicates). Case–control association was assessed descriptively using both unadjusted and age- and sex-adjusted additive logistic regression, with the adjusted model as primary. Results: Reference frequencies were available for 1533 of 1939 variants (1363 at MAF ≥ 0.05). Frequencies clustered with South Asian (FST = 0.077, 95% CI 0.065–0.089) and European panels (FST = 0.086, 95% CI 0.072–0.099), which were indistinguishable (ΔFST = −0.010, 95% CI −0.022 to 0.003) and more divergent from East Asian and African panels. Divergence from the European and South Asian panels was unrelated to nominal association status (Mann–Whitney *p* = 0.99 and 0.57). In the primary age- and sex-adjusted analysis, no variant survived multiple-testing correction (minimum Bonferroni-adjusted and false-discovery-rate q = 0.28), consistent with the unadjusted sensitivity analysis. Conclusions: This study provides the first systematic allele-frequency reference for cardiometabolic risk loci in an Uzbek cohort from Tashkent. The profile is closest to South Asian and European panels and identifies PRS from these ancestries as priority candidates for future local validation in Central Asian settings. The case–control analysis was underpowered and is reported descriptively.

## 1. Introduction

The global burden of type 2 diabetes (T2D) continues to rise, and Central Asia is among the regions with the steepest trajectories [1,2]. Uzbekistan ranks second worldwide in the rate of growth of age-standardised T2D incidence; however, the genetic architecture of the disease in this population has been examined in only a small number of candidate studies [3].

Large multi-ancestry studies have transformed the genetic map of T2D [4,5,6]. The most recent effort, based on 2,535,601 individuals of whom 39.7% were of non-European ancestry, identified 1289 independent association signals mapping to 611 loci [4]. However, the reference resources on which such analyses depend remain heavily skewed: individuals of European descent continue to dominate genomic databases [7,8], and PRS transfer poorly across ancestries, with predictive accuracy declining as genetic distance from the discovery cohort increases [9,10,11]. The practical consequence is that a PRS validated in Europeans cannot be assumed to perform equivalently in an unstudied population.

Central Asia occupies a distinctive position in this landscape. The populations of the region carry admixture signatures accumulated across millennia of movement along the Silk Road, with contributions from both western and eastern Eurasian sources [12]. No Central Asian population is represented among the 1000 Genomes Project Phase 3 reference populations [13], so the region has no direct reference panel. For any group in this position, the first practical question is not which variants are associated with disease locally, but simply what the allele frequencies are at loci already known to matter, and which existing reference panel they most closely resemble. This question can be answered with modest sample sizes, because frequency estimation does not require the statistical power that association discovery demands.

We therefore set out to characterise effect-allele frequencies at 1939 cardiometabolic candidate variants in an Uzbek cohort from Tashkent, to quantify the divergence of this frequency profile from each continental 1000 Genomes superpopulation, and to identify which reference ancestry is the most plausible starting point for future PRS evaluation. A case–control association analysis is also reported for context; however, the sample size does not support discovery claims.

## 2. Materials and Methods

### 2.1. Participants and Sample Provenance

The study included 171 unrelated adults recruited in Tashkent, Uzbekistan: 131 individuals with type 2 diabetes (T2D) and 40 individuals without diabetes. T2D was defined according to World Health Organization criteria (fasting plasma glucose ≥ 7.0 mmol/L or 2 h oral glucose tolerance test ≥ 11.1 mmol/L) [14] and confirmed by an endocrinologist. Participants with T2D were recruited consecutively at the Republican Specialized Scientific and Practical Medical Center of Cardiology and at the Center of Endocrinology (Tashkent). Inclusion criteria for cases were physician-confirmed type 2 diabetes, adult onset, and Uzbek nationality (age range 35–75 years, mean 56.3); exclusion criteria were type 1 diabetes and non-Uzbek nationality. Controls were adult volunteers (age ≥ 40 years, Uzbek nationality) without a personal or first-degree family history of diabetes who had undergone clinical examination. Islet autoantibody and C-peptide testing were not performed, so type 1 diabetes and maturity-onset diabetes of the young could not be excluded by laboratory criteria (see Limitations). All participants were recruited from a single metropolitan area, so the sample was not stratified across the regions of Uzbekistan.

The study was conducted in accordance with the Declaration of Helsinki and approved by the Ethical Committee of the Ministry of Health of the Republic of Uzbekistan (Protocol No. 1/8, 23 January 2025). Written informed consent was obtained from all participants.

### 2.2. Genotyping and Variant Selection

Genotyping was performed using a custom targeted enrichment panel ordered from Agilent Technologies, Santa Clara, CA, USA (SureSelect), not a genome-wide genotyping array. The panel was designed around curated candidate SNPs previously associated with T2D, cardiovascular disease and lipid traits in public GWAS resources; it was not designed as an ancestry-informative or genome-wide marker set. Probes were selected for the corresponding genomic intervals using Agilent design tools. In addition to the originally targeted sites, the capture assay recovered flanking variants within the enriched regions. The working genotype matrix contained 4588 called variants across 171 samples. Because the assay was restricted to preselected disease-related candidate regions, genome-wide ancestry inference (for example, principal components or ADMIXTURE) was not possible, and no ancestry covariates were used at any stage of the analysis. The final analysis dataset comprised 171 uniquely identified participants after correction of sample-labelling inconsistencies identified during data curation (two mislabelled genotype records were corrected; no participant was excluded).

### 2.3. Quality Control

Quality control proceeded as follows. Of the 4588 input variants, 118 were monomorphic and were removed; Hardy–Weinberg equilibrium was assessed in the full sample and variants with *p* < 0.05 were removed (*n* = 330)—a conservative threshold chosen to minimise genotyping artefacts in a modestly sized cohort (control-only HWE filtering was not applied because the control group was small, *n* = 40)—and variants with a minor allele count below 10 were removed. This left 1947 variants passing quality control. Eight of these sat at the MAC inclusion threshold (MAC = 10) with the minor allele absent from controls and were not carried into the analysis set, leaving 1939 variants. Population-genetic analyses used this set of 1939 variants; the additive association model used the 1936 biallelic markers among them. No selection on association *p*-value was applied at any point. To confirm that the population-genetic results did not depend on the HWE strategy, we repeated the analysis (i) with no HWE filter and (ii) with an exact HWE test applied in controls only; FST values changed by ≤0.003 and the three-tier structure was preserved (Supplementary Table S4).

### 2.4. Reference Frequencies and Harmonisation

Allele frequencies for the four 1000 Genomes Project Phase 3 superpopulations selected for this analysis (EUR, SAS, EAS, and AFR) [13] were retrieved programmatically via the Ensembl REST API [15]. The admixed American (AMR) superpopulation was not included because it aggregates populations with heterogeneous recent admixture histories and was not an appropriate comparator for the specific objective of positioning the cohort relative to broad African and Eurasian references. We used 1000 Genomes Phase 3 as the primary reference because it provides publicly accessible, strand-harmonisable allele frequencies across matched superpopulation panels; larger resources such as gnomAD [16] and GenomeAsia 100K [17] were not used for the formal FST comparisons because they do not provide equivalent discrete superpopulation definitions (see Section 4). For each variant, the frequency of the same effect allele as defined in the Uzbek cohort was extracted; where the reported allele corresponded to the opposite DNA strand, the frequency was reassigned to the complementary allele. Strand-ambiguous variants (A/T and C/G) with an effect-allele frequency between 0.35 and 0.65 were excluded because the strand cannot be resolved unambiguously for such sites. Reference frequencies were available for 1533 of 1939 variants; the remaining 405 were absent from 1000 Genomes Phase 3 or corresponded to merged or retired rs identifiers. Analyses were restricted to variants with an Uzbek minor allele frequency ≥ 0.05 (*n* = 1363) unless stated otherwise.

### 2.5. Population-Genetic Analysis

Pairwise differentiation was quantified using the Hudson estimator of FST with a sample-size correction, aggregated as a ratio of averages across loci, which is the recommended approach when sample sizes differ substantially between groups [18]. Nei’s standard genetic distance was computed for the same variant set [19]. Confidence intervals and pairwise contrasts were obtained using non-parametric bootstrap resampling over loci (1000 replicates). To assess whether the frequency profile was influenced by within-cohort variant selection, per-locus FST was compared between variants reaching nominal association significance and the remainder using the Mann–Whitney U test. Because the panel includes variants captured within the same enrichment intervals, we characterised linkage disequilibrium (LD) directly from the individual genotypes: of the 1363 analysed variants, 818 pairs showed r^2^ > 0.2 (697 variants), forming 910 single-linkage LD blocks. To ensure that confidence intervals were not anti-conservative through non-independence, the differentiation analysis was repeated on an LD-pruned set (greedy pruning at r^2^ > 0.2; 931 variants) and using a block bootstrap that resampled LD blocks rather than individual SNPs (1000 replicates); both are summarised in Supplementary Table S4 together with the Hardy–Weinberg and controls-only sensitivities.

### 2.6. Association Analysis

Case–control association was tested by additive logistic regression of disease status on minor-allele dosage (0/1/2). The primary model was adjusted for age and sex; the unadjusted model is reported as a sensitivity analysis. For each variant the effect allele is the cohort minor allele, which is also the coded (dosage) allele in the regression and the allele to which reference frequencies were harmonised, so “effect allele” and “minor allele” denote the same allele throughout. Odds ratios and 95% confidence intervals were obtained by exponentiating the regression coefficients. Multiple-testing correction was applied across the 1936 biallelic variants using the Bonferroni and Benjamini–Hochberg false discovery rate procedures. The genomic inflation factor λGC was computed from the median test statistic. Statistical power was evaluated analytically for representative minor allele frequencies at the corrected significance threshold. Analyses were performed in Python 3.12.3 using statsmodels 0.15.0, NumPy 2.4.4, SciPy 1.17.1 and pandas 3.0.2. Variants exhibiting complete or quasi-complete separation were fitted with Firth’s penalised-likelihood logistic regression to obtain finite effect estimates; multiallelic markers were excluded from the additive model.

### 2.7. Public Annotation of Assayed Variants

To place the assayed variants in the broader literature, all 1939 quality-controlled rs identifiers were cross-referenced to the NHGRI-EBI GWAS Catalog (ontology-annotated association release) [20] and annotated with the Ensembl Variant Effect Predictor gene and consequence fields [21]. For each variant, we recorded GRCh38 coordinates, the most severe VEP consequence, overlapping gene symbols, and—where a GWAS Catalog association exists—trait labels with PubMed identifiers. Two rs identifiers do not map to GRCh38. These annotations are provided as Supplementary Table S3 and are intended as a lookup resource rather than as evidence of association in the present cohort.

## 3. Results

### 3.1. Cohort Characteristics and Variant Quality Control

The baseline characteristics of the 171 participants are summarised in Table 1. Cases were older than controls and had higher body mass index, fasting plasma glucose, and glycated haemoglobin, as expected for a clinically ascertained diabetes cohort. Of the 4588 genotyped variants, 1947 passed quality control; eight variants at the MAC inclusion threshold (MAC = 10; minor allele absent from controls) were not carried forward, leaving 1939 analysed (Table 2).

### 3.2. Effect-Allele Frequency Profile

**Figure 1:**
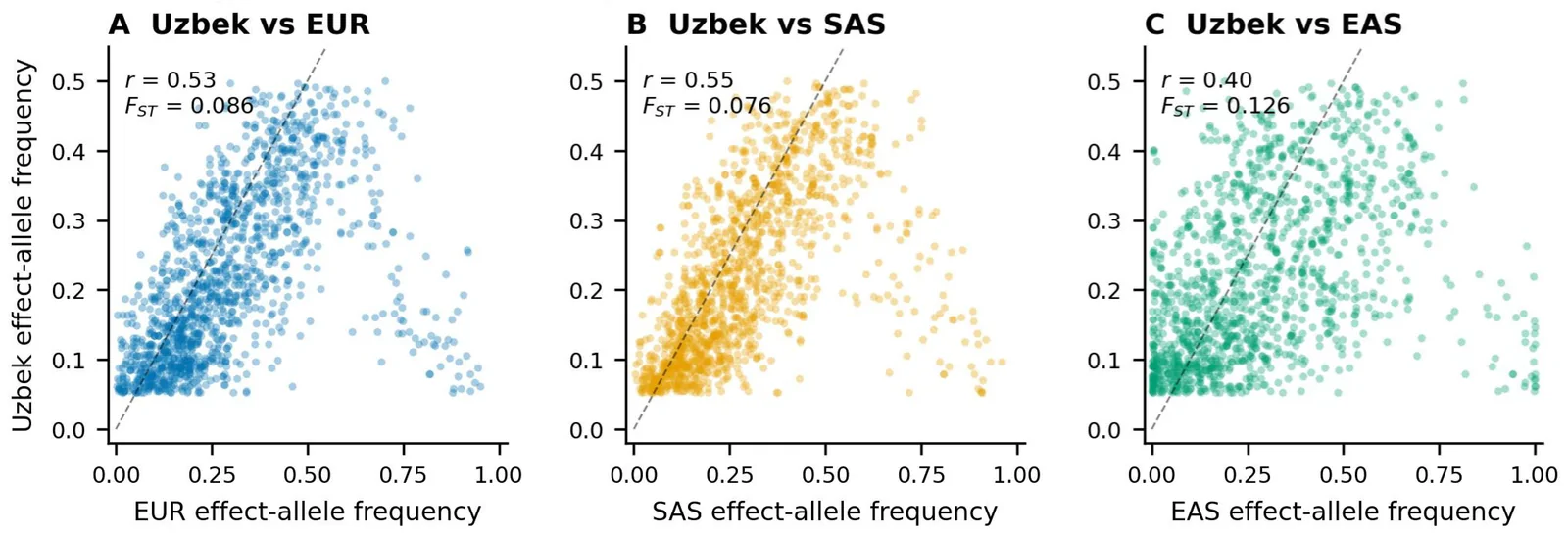
Effect-allele frequencies at cardiometabolic candidate loci. Effect-allele frequency in the Tashkent Uzbek cohort plotted against European (**A**), South Asian (**B**) and East Asian (**C**) 1000 Genomes Phase 3 frequencies for 1363 variants with minor allele frequency ≥ 0.05. Frequencies are harmonised to the same effect allele in all populations. The dashed line indicates identity. Pearson correlation and Hudson FST are inset in each panel.

Effect-allele frequencies in the Tashkent Uzbek cohort correlated strongly with all three Eurasian reference panels, most closely with South Asian and European frequencies (Figure 1). The distribution of frequencies was continuous across the allele-frequency spectrum, with no evidence of systematic truncation or platform-specific artefact. Because the cohort is predominantly case-ascertained (131 T2D cases and 40 controls), cohort-wide allele frequencies may be modestly shifted relative to those of an unselected population sample; we therefore present them as a practical reference for this clinically recruited metropolitan cohort rather than as census frequencies for Uzbekistan as a whole.

Harmonised effect-allele frequencies for the 1363 variants with matching 1000 Genomes Phase 3 data and Uzbek MAF ≥ 0.05 are provided as Supplementary Table S1 and constitute the principal reference output of this study. Association statistics for the 1936 biallelic variants are provided in Supplementary Table S2, and publicly reported phenotype associations and gene annotations for the 1939 assayed variants are summarised in Supplementary Table S3. To confirm that enrichment for T2D did not distort the frequency profile, allele frequencies were also estimated in the 40 controls alone. These correlated strongly with the full-cohort estimates (Pearson r = 0.94), and the three-tier structure was preserved (controls-only FST: SAS 0.076 (95% CI 0.064–0.088), EUR 0.081 (0.069–0.095), EAS 0.130 (0.116–0.146), AFR 0.177 (0.163–0.192)), with South Asian remaining closest (FST = 0.076) followed by European (FST = 0.081; Supplementary Table S4).

### 3.3. Population Differentiation

**Figure 2:**
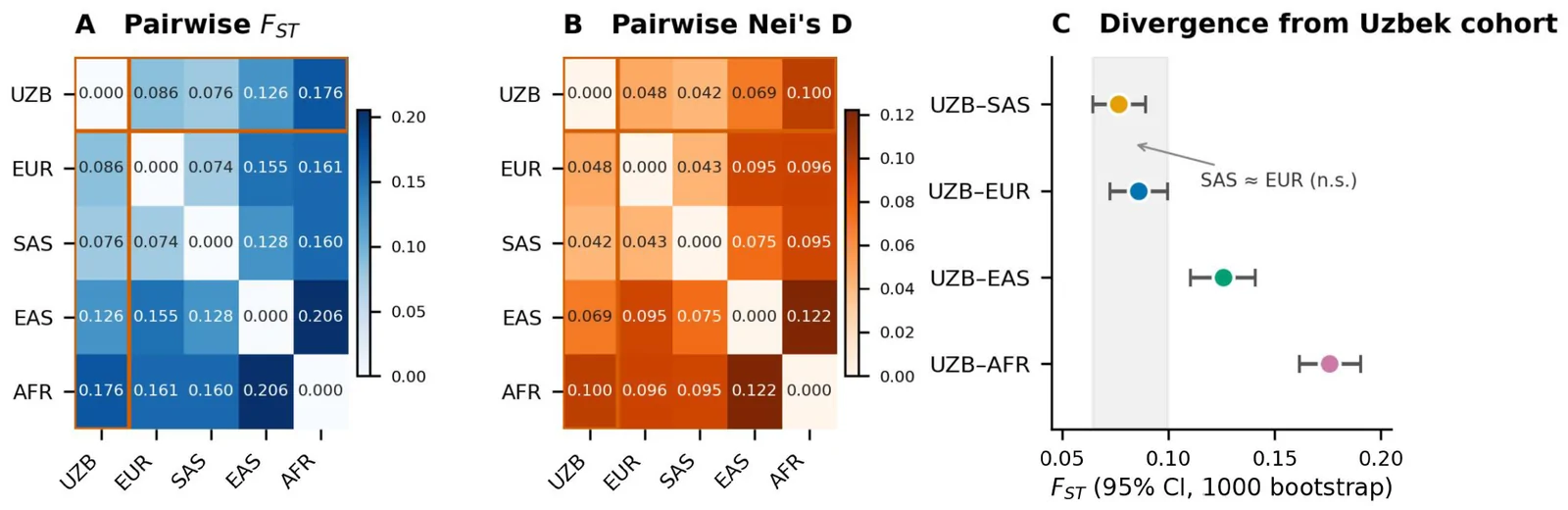
Population differentiation at cardiometabolic candidate loci. (**A**) Pairwise Hudson FST matrix across the Tashkent Uzbek cohort and four continental 1000 Genomes superpopulations; the Uzbek row and column are outlined. (**B**) Corresponding matrix of Nei’s standard genetic distance. (**C**) Divergence of each reference panel from the Uzbek cohort with 95% confidence intervals from 1000 bootstrap replicates over loci; the shaded band indicates the overlap between the South Asian and European estimates, which are not distinguishable (ΔFST = −0.010, 95% CI −0.022 to 0.003). In panel (**C**), n.s. indicates a non-significant SAS–EUR contrast (the ΔFST 95% CI includes zero). Colours in panel (**C**) match panels (**A**,**B**): gold, SAS; blue, EUR; green, EAS; magenta, AFR.

Pairwise differentiation placed the Uzbek cohort closest to the South Asian panel (FST = 0.077, 95% CI 0.065–0.089; Nei’s D = 0.042) and the European panel (FST = 0.086, 95% CI 0.072–0.099; Nei’s D = 0.048), followed by the East Asian (FST = 0.126, 95% CI 0.110–0.142) and African panels (FST = 0.176, 95% CI 0.162–0.189) (Figure 2, Table 3).

The same ordering was obtained after accounting for linkage among SNPs. On the LD-pruned set, FST values were essentially unchanged (UZB–SAS 0.077, 95% CI 0.062–0.095; UZB–EUR 0.087, 0.071–0.105; UZB–EAS 0.132, 0.113–0.153; UZB–AFR 0.174, 0.156–0.194), and block bootstrap resampling of LD blocks preserved the key contrasts (UZB–SAS vs. UZB–EUR ΔFST = −0.010, 95% CI −0.023 to +0.003, not significant; UZB–EUR vs. UZB–EAS ΔFST = −0.040, 95% CI −0.055 to −0.025, significant).

Bootstrap contrasts showed that the South Asian and European panels were not distinguishable from one another with respect to distance from the Uzbek cohort (ΔFST = −0.010, 95% CI −0.022 to 0.003). Both were, however, significantly closer than the East Asian panel (South Asian versus East Asian: ΔFST = −0.049, 95% CI −0.065 to −0.034; European versus East Asian: ΔFST = −0.040, 95% CI −0.056 to −0.025), and the East Asian panel, in turn, was significantly closer than the African panel (ΔFST = −0.050, 95% CI −0.067 to −0.033). The frequency profile of the cohort therefore resolves into three statistically separable tiers: South Asian and European together, then East Asian, then African.

As an internal check, differentiation was also computed between pairs of reference panels, where the expected ordering is established. The observed ordering reproduced the known relationships among continental groups exactly (European–South Asian < South Asian–East Asian < European–East Asian < European–African < East Asian–African), confirming that the analytical pipeline behaves as expected on this variant set.

### 3.4. Differentiation Is Independent of Association Status

**Figure 3:**
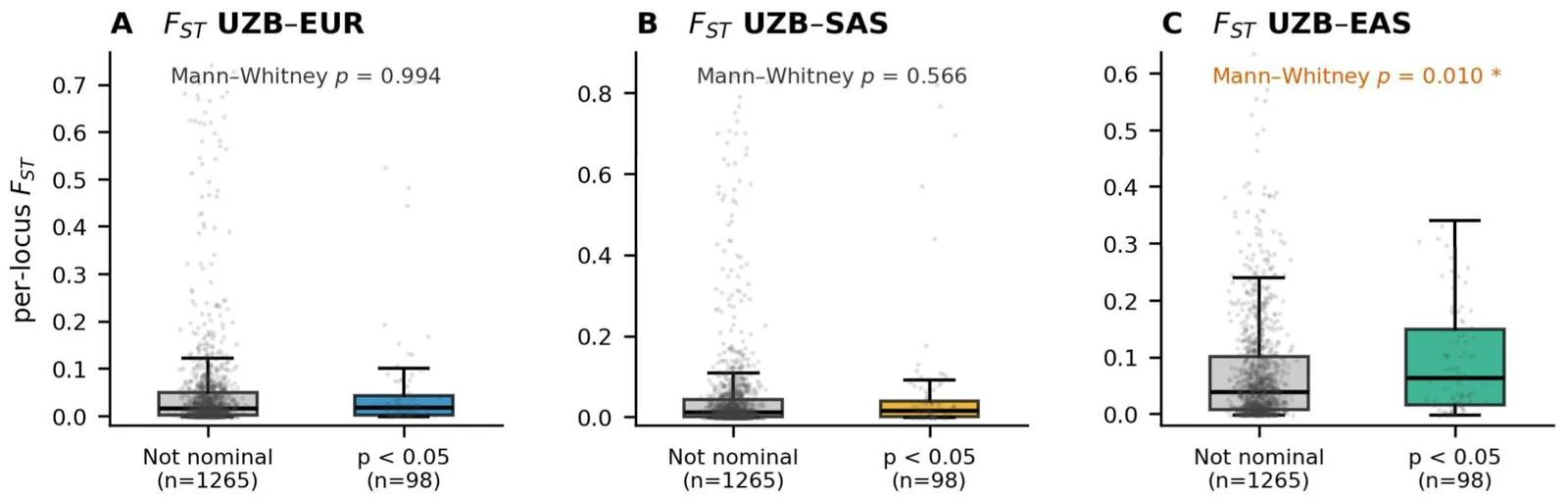
Population differentiation by nominal association status. Per-locus Hudson FST between the Uzbek cohort and European (**A**), South Asian (**B**) and East Asian (**C**) reference panels, compared between variants reaching nominal association significance (*p* < 0.05) and all remaining variants. Boxes show the median and interquartile range; individual loci are overlaid; Mann–Whitney *p*-values are inset (an asterisk marks *p* < 0.05; orange inset text denotes *p* < 0.05, black inset text denotes non-significant comparisons). No difference is seen for the European or South Asian panels; a modest significant difference for the East Asian panel does not affect the main conclusion, as the East Asian panel is the most divergent from the Uzbek cohort in all analyses.

Because population-genetic statistics computed on variants preselected for case–control differences can be vulnerable to the winner’s curse, we tested whether nominally associated variants showed elevated differentiation. Per-locus FST between the Uzbek cohort and the European and South Asian panels did not differ between variants with nominal *p* < 0.05 and the remainder (Mann–Whitney *p* = 0.99 and *p* = 0.57, respectively; Figure 3). For the East Asian panel a modest but significant difference was observed (*p* = 0.010). Because the East Asian panel is in all analyses the most divergent from the Uzbek cohort, this does not affect the conclusion that Uzbek frequencies are closest to South Asian and European panels. The frequency profile underlying our main result is therefore not an artefact of within-cohort selection.

### 3.5. Case–Control Association (Descriptive)

**Figure 4:**
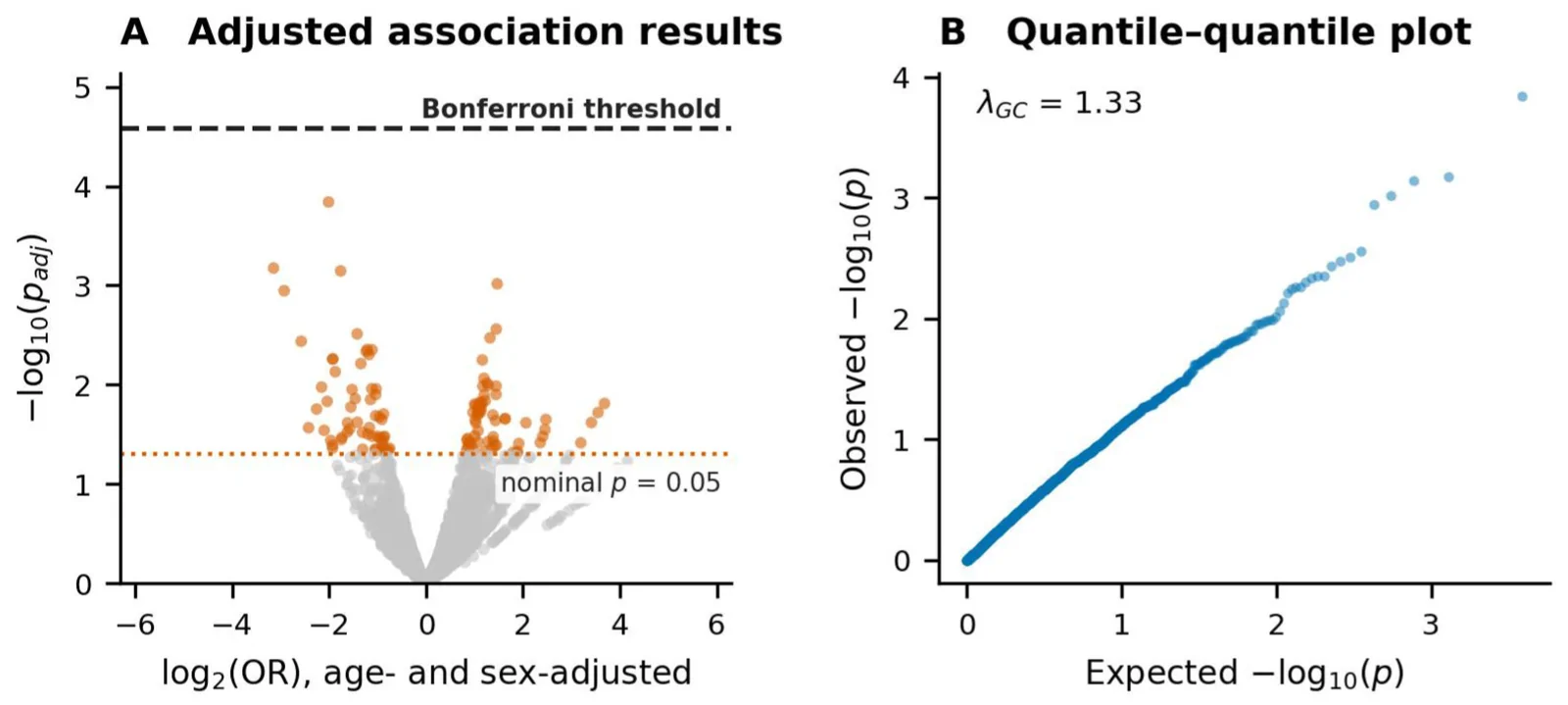
Candidate-locus association analysis, age- and sex-adjusted. (**A**) Effect size (adjusted log_2_ odds ratio) versus significance for the 1936 analysed variants. The dotted line marks nominal *p* = 0.05 and the dashed line the Bonferroni threshold (*p* = 2.58 × 10^−5^); no variant exceeds the corrected threshold. Variants with nominal adjusted *p* < 0.05 are highlighted. For display, variants with |log_2_(OR)| > 6 (near-complete allelic separation at low minor allele counts) are omitted from the horizontal axis; however, all 1936 analysed variants are included in panel B. (**B**) Quantile–quantile plot of observed against expected *p*-values, with genomic inflation factor λGC = 1.33 inset.

In the primary age- and sex-adjusted analysis, 120 of the 1936 biallelic variants tested reached nominal significance (*p* < 0.05). No variant remained significant after correction for multiple testing (minimum Bonferroni-adjusted and Benjamini–Hochberg q = 0.28; minimum raw adjusted *p* = 1.4 × 10^−4^). Results were consistent in the unadjusted sensitivity analysis (129 nominal; minimum false-discovery-rate q = 0.12). The strongest signal was rs13376333 (KCNN3), which remained the top variant after adjustment (adjusted OR = 0.25, 95% CI 0.12–0.51, *p* = 1.4 × 10^−4^; unadjusted OR = 0.25, *p* = 5.9 × 10^−5^). Fourteen variants showing complete or quasi-complete separation were fitted with Firth’s penalised-likelihood logistic regression to obtain finite estimates. Three additional multiallelic markers were excluded from the additive model, so that 1936 of the 1939 tested variants were analysed. These association results are summarised in Figure 4.

A power analysis showed that, at the Bonferroni threshold (α = 2.6 × 10^−5^) with 131 cases and 40 controls, the minimum detectable odds ratio at 80% power was approximately 3.6 at MAF 0.50, 5.0 at MAF 0.20, 8.6 at MAF 0.10 and 19 at MAF 0.05; the study is therefore powered only to detect very large effects. The genomic inflation factor was λGC = 1.33 (median χ^2^ statistic divided by its expected value); in a panel enriched for previously associated loci, inflation of this magnitude is expected. Adjusted and unadjusted results for all variants are provided in Supplementary Table S2. Given the sample size, these results are reported descriptively, and no individual variant is interpreted as disease-associated in this cohort.

## 4. Discussion

This study provides the first systematic description of effect-allele frequencies at cardiometabolic risk loci in an Uzbek cohort from Tashkent. The central observation is that the frequency profile sits closest to South Asian and European reference panels, which cannot be separated from one another (ΔFST = −0.010, 95% CI −0.022 to 0.003), and is progressively and significantly more distant from East Asian and African panels. This tiered structure is consistent with the documented admixture history of inner Eurasia, in which Central Asian groups carry ancestry from both western and eastern sources with a predominance of the former [12].

These frequency relationships support future evaluation of PRS derived from European or South Asian cohorts in Central Asian settings; they do not demonstrate transferability [9,10,11]. Because the two panels were indistinguishable, the data do not favour one over the other, and any application would require independent local validation.

A similar picture has been reported in other under-represented populations. In the Qatar Biobank, a whole-genome-sequencing study of 11,436 individuals replicated the majority of known T2D loci and found that the effect sizes and allele frequencies of replicated SNPs generally concurred with European estimates, while a multi-ancestry polygenic score outperformed a European-only score [22]. Our frequency-based results extend this picture to Central Asia: the close correspondence of Uzbek effect-allele frequencies with South Asian and European panels suggests that PRS incorporating these ancestries are the most plausible starting point for a future locally validated score, whereas an exclusively East Asian-derived score would require separate local evaluation. Clinically, such ancestry-aware PRS calibration is a prerequisite before polygenic risk information could be used to prioritise screening or preventive care in Uzbek patients.

An important methodological caveat concerns the absolute magnitude of the differentiation estimates. Because the variant panel consists exclusively of loci previously associated with cardiometabolic traits in predominantly European GWAS, it is not a neutral or ancestry-informative marker set, and genome-wide stratification cannot be assessed using these data. When the same statistics were computed between pairs of reference panels, the values exceeded published genome-wide estimates by approximately 1.1- to 2.9-fold [18], while preserving the correct ordering. Absolute FST and Nei’s D values should therefore be interpreted as panel-specific quantities describing these loci, not as estimates of genome-wide population structure. The relative ordering, which is the basis of our conclusions, is robust.

Formal FST comparisons were restricted to 1000 Genomes Project Phase 3 superpopulations to enable matched, publicly reproducible distance estimates [13]. Complementary resources such as gnomAD [16] and GenomeAsia 100K [17] offer larger sample sizes and broader geographic coverage, and will be useful for variant-level lookups as Central Asian representation improves; they were not used here as primary FST comparators because they lack the same discrete superpopulation definitions.

The case–control component warrants little interpretation: with 131 cases and 40 controls, the analysis has limited power, and no variant survived correction for multiple testing. Nominal signals at several loci are directionally consistent with prior reports but cannot be distinguished from chance at the present sample size. This cohort shares participants with a previous report from our group in which six candidate variants were genotyped by RT-PCR [3]; the studies use different panels and approaches, and the present results should not be regarded as independent replication.

### 4.1. Limitations

The study has several limitations. First, the sample is small (*N* = 171) and the control group in particular is limited to 40 individuals; no association survived correction for multiple testing, and association results are therefore reported descriptively. Second, all participants were recruited in Tashkent, so the sample may not capture regional genetic substructure within Uzbekistan; results should be interpreted as applying to this metropolitan cohort rather than to the country as a whole. Third, type 1 diabetes and maturity-onset diabetes of the young were not excluded by autoantibody or C-peptide testing; case definition relied on clinical criteria alone. Fourth, genotyping used a targeted candidate panel designed for disease-associated loci rather than ancestry inference, so genome-wide population structure could not be assessed and association models were adjusted for age and sex but not for genome-wide ancestry; frequency comparisons describe the assayed loci only. Cases and controls differed in body mass index, and because the panel includes obesity- and lipid-associated loci, the nominal association signals may partly reflect correlates of adiposity; this is a further reason why the case–control results are treated as descriptive only. Fifth, cohort-wide allele frequencies are estimated in a predominantly case-ascertained sample and may differ from those in an unselected population. Figure 3 indicates that nominal association status does not drive the differentiation pattern, but control-only or population-based estimates would still be preferable. Sixth, no Central Asian population is represented in 1000 Genomes Phase 3, and other large resources (for example, gnomAD [16] or GenomeAsia 100K [17]) were not used for the primary FST analysis. Finally, the cohort shares participants with a previously published series [3] and the present study does not constitute independent replication.

### 4.2. Future Directions

Extending genotyping to a genome-wide array or whole-genome sequencing in a larger, regionally stratified Uzbek sample would permit direct estimation of population structure and local ancestry, and would support formal testing of PRS performance. Inclusion of Central Asian reference panels in international resources remains a prerequisite for equitable genomic medicine in the region.

## 5. Conclusions

We report effect-allele frequencies at 1939 cardiometabolic candidate variants in an Uzbek cohort from Tashkent. This clinically recruited sample has no counterpart in existing genomic databases and provides the first local frequency reference. The frequency profile is closest to South Asian and European panels, which are indistinguishable from one another, and significantly more distant from East Asian and African panels; this ordering is independent of within-cohort association status. These results support the future evaluation of European- or South Asian-derived polygenic scores in Central Asian settings, subject to local validation. The case–control analysis is underpowered and is reported descriptively: no variant remained significant after correction for multiple testing.

## Figures and Tables

**Table 1:** Baseline characteristics of the study cohort (*N* = 171).

Characteristic	T2D Cases (*n* = 131)	Controls (*n* = 40)	*p*-Value
Age, years (mean ± SD)	56.3 ± 9.8	48.7 ± 11.4	<0.001
Female, *n* (%)	82 (62.6)	21 (52.5)	0.27
Body mass index, kg/m^2^	30.1 ± 5.2	26.4 ± 4.1	<0.001
Fasting plasma glucose, mmol/L	9.4 ± 3.1	5.1 ± 0.5	<0.001
HbA1c, %	8.2 ± 1.9	5.5 ± 0.3	<0.001
T2D duration, years	7.2 ± 5.8	—	—
On metformin, *n* (%)	112 (85.5)	—	—

Values are mean ± SD unless otherwise indicated. Group comparisons were performed using Student’s *t*-test (for continuous variables) or Fisher’s exact test (for sex). Clinical variables are summarised from the study clinical database at enrolment for the 171 genotyped participants. All participants were recruited in Tashkent. Summary statistics are not intended for direct numerical comparison with other published extracts from overlapping clinical series that may use different inclusion windows or summary metrics.

**Table 2:** Variant quality control.

Step	Criterion	Variants
Input	All genotyped variants	4588
Monomorphic	Only one allele in cohort; removed	−118
Hardy–Weinberg equilibrium	Removed at *p* < 0.05 (full sample)	−330
Minor allele count	Removed at MAC < 10	−2193
Quality control passed		**1947**
Case–control separation	MAC = 10 and control MAF = 0; not carried forward	−8
Analysed	Carried into analyses	**1939**
With 1000 Genomes reference data		1533
Analysis set (MAF ≥ 0.05)		1363
Biallelic association model	3 multiallelic excluded; 14 Firth	1936

MAC, minor allele count; MAF, minor allele frequency. Bold counts mark the QC-passed set (1947) and the analysis set carried forward (1939). Eight QC-passed variants were not carried forward: each had MAC = 10, with the minor allele observed only among cases (control MAF = 0): rs115734262, rs116755303, rs1264631877, rs146387099, rs1767570064, rs201398141, rs372798351 and rs531675656. Of the 1939 variants carried forward, three were multiallelic and were excluded from the additive association model (*n* = 1936). Fourteen other variants with the same qualitative pattern but higher minor-allele counts (MAC 11–18) were retained and fitted with Firth’s penalised-likelihood logistic regression.

**Table 3:** Pairwise population differentiation at 1363 cardiometabolic candidate loci.

Comparison	FST (Hudson)	95% CI	Nei’s D
Uzbek–South Asian	0.077	0.065–0.089	0.042
Uzbek–European	0.086	0.072–0.099	0.048
Uzbek–East Asian	0.126	0.110–0.142	0.069
Uzbek–African	0.176	0.162–0.189	0.099
European–South Asian	0.074	—	0.043
South Asian–East Asian	0.128	—	0.075
European–East Asian	0.155	—	0.095
European–African	0.161	—	0.096
East Asian–African	0.206	—	0.122

Confidence intervals from 1000 bootstrap replicates over loci. Reference–reference comparisons are shown for calibration; their ordering reproduces established continental relationships. Absolute values are elevated relative to genome-wide estimates because the panel comprises trait-associated rather than neutral loci and should be interpreted as panel-specific quantities.

## Data Availability

The genotype dataset is deposited at Figshare (https://doi.org/10.6084/m9.figshare.32287434). Harmonised allele frequencies for variants with 1000 Genomes Phase 3 matches and Uzbek MAF ≥ 0.05 (*n* = 1363) are provided as Supplementary Table S1; association results for 1936 biallelic variants are provided as Supplementary Table S2; phenotype and gene annotations for the 1939 assayed variants are provided as Supplementary Table S3; FST sensitivity analyses are provided as Supplementary Table S4. Analysis code is available from the corresponding author on reasonable request.

## Supplementary Materials

The following supporting information can be downloaded at: https://www.mdpi.com/article/10.3390/genes17091117/s1, Supplementary Table S1: Harmonised effect-allele frequencies. Supplementary Table S2: (a) Association results—age- and sex-adjusted (primary model); (b) Association results—unadjusted (sensitivity). Supplementary Table S3: Functional and phenotypic annotation of the 1939 analysed variants. Supplementary Table S4: Sensitivity analyses of population differentiation.
